# The Role of Iron in Friedreich’s Ataxia: Insights From Studies in Human Tissues and Cellular and Animal Models

**DOI:** 10.3389/fnins.2019.00075

**Published:** 2019-02-18

**Authors:** José Vicente Llorens, Sirena Soriano, Pablo Calap-Quintana, Pilar Gonzalez-Cabo, María Dolores Moltó

**Affiliations:** ^1^Department of Genetics, Faculty of Biological Sciences, University of Valencia, Valencia, Spain; ^2^Unit for Psychiatry and Neurodegenerative Diseases, Biomedical Research Institute INCLIVA, Valencia, Spain; ^3^Department of Molecular and Human Genetics, Baylor College of Medicine, Houston, TX, United States; ^4^Jan and Dan Duncan Neurological Research Institute, Texas Children’s Hospital, Houston, TX, United States; ^5^Department of Physiology, Faculty of Medicine and Dentistry, University of Valencia, Valencia, Spain; ^6^Center of Biomedical Network Research on Rare Diseases CIBERER, Valencia, Spain; ^7^Associated Unit for Rare Diseases INCLIVA-CIPF, Biomedical Research Institute INCLIVA, Valencia, Spain; ^8^Center of Biomedical Network Research on Mental Health CIBERSAM, Valencia, Spain

**Keywords:** Friedreich’s ataxia, frataxin, iron, animal models, oxidative stress, lipid deregulation, iron chelators

## Abstract

Friedreich’s ataxia (FRDA) is a rare early-onset degenerative disease that affects both the central and peripheral nervous systems, and other extraneural tissues, mainly the heart and endocrine pancreas. This disorder progresses as a mixed sensory and cerebellar ataxia, primarily disturbing the proprioceptive pathways in the spinal cord, peripheral nerves and nuclei of the cerebellum. FRDA is an inherited disease with an autosomal recessive pattern caused by an insufficient amount of the nuclear-encoded mitochondrial protein frataxin, which is an essential and highly evolutionary conserved protein whose deficit results in iron metabolism dysregulation and mitochondrial dysfunction. The first experimental evidence connecting frataxin with iron homeostasis came from *Saccharomyces cerevisiae*; iron accumulates in the mitochondria of yeast with deletion of the frataxin ortholog gene. This finding was soon linked to previous observations of iron deposits in the hearts of FRDA patients and was later reported in animal models of the disease. Despite advances made in the understanding of FRDA pathophysiology, the role of iron in this disease has not yet been completely clarified. Some of the questions still unresolved include the molecular mechanisms responsible for the iron accumulation and iron-mediated toxicity. Here, we review the contribution of the cellular and animal models of FRDA and relevance of the studies using FRDA patient samples to gain knowledge about these issues. Mechanisms of mitochondrial iron overload are discussed considering the potential roles of frataxin in the major mitochondrial metabolic pathways that use iron. We also analyzed the effect of iron toxicity on neuronal degeneration in FRDA by reactive oxygen species (ROS)-dependent and ROS-independent mechanisms. Finally, therapeutic strategies based on the control of iron toxicity are considered.

## Introduction

Nicholaus Friedreich, a German neurologist among a family of professors of medicine, had described a new disease entity in successive papers published between 1863 and 1877 (Koeppen, 2013). The new disease, named later Friedreich’s ataxia (FRDA), was characterized by degenerative atrophy of the posterior columns of the spinal cord, leading to the development of progressive ataxia, sensory loss and muscle weakness in patients and was associated with cardiomyopathy in many cases. Clear diagnostic criteria for FRDA were established by Anita Harding in the 1980s (Harding, 1981), who specified the hereditary nature of the disease with an autosomal recessive pattern, an onset during puberty, progressive ataxia of the limbs and gait with the absence of tendon reflexes in the legs, extensor plantar responses, and dysarthria. In 1996, the identification of the causative gene of FRDA and expansion of a trinucleotide repeat as the most frequent mutation (Campuzano et al., 1996) led to the development of a molecular tool for a definitive diagnosis of the disease. It was crucial to address the differential diagnosis with respect to other peripheral neuropathies and ataxias with similar clinical features, as well as the clinical heterogeneity in FRDA, including atypical phenotypes (Bidichandani and Delatycki, 2017). Moreover, the availability of this molecular approach allowed for appropriate genetic counseling.

### Epidemiological, Pathophysiological and Molecular Aspects of FRDA

Friedreich’s ataxia (OMIM #229300; ORPHA:95) is a rare neurodegenerative disease affecting 1:20,000–1:50,000 people of Indo-European and North African descendent (Vankan, 2013), and with a carrier frequency of 1:60–1:100, FRDA is the most frequently inherited ataxia in these populations. The neurological signs and symptoms in FRDA are the results of pathological changes that disturb both the central and peripheral nervous systems (NSs) (reviewed in Marmolino, 2011; Koeppen and Mazurkiewicz, 2013). The dorsal root ganglia (DRG) are particularly vulnerable and have been identified as the first site of neuropathology with the degeneration of the large sensory neurons and posterior columns, leading to the loss of position and vibration senses. Next, affectation of the corticospinal and spinocerebellar tracts of the spinal cord occurs, with loss of myelinated fibers. In the cerebellum, dentate nuclei (DN) also show progressive atrophy. Cerebral abnormalities are being progressively reported with the application of neuroimaging techniques (Selvadurai et al., 2018). Non-neurological phenotypes of FRDA mainly include hypertrophic cardiomyopathy, which is the leading cause of mortality (Raman R. et al., 2011; Payne and Wagner, 2012; Weidemann et al., 2013; Koeppen et al., 2015). The heart typically maintains adequate systolic function (Kipps et al., 2009) in FRDA patients who develop a severe hypertrophic cardiomyopathy until shortly before death (Rajagopalan et al., 2010). Additionally, diabetes mellitus and skeletal abnormalities are also present in this disease (Parkinson et al., 2013; Bidichandani and Delatycki, 2017).

Friedreich’s ataxia is caused by loss-of-function mutations in the frataxin gene (*FXN*) (Campuzano et al., 1996). The most common mutation appears in homozygosis in most patients and consists of an abnormally expanded GAA trinucleotide repeat (usually between 600 and 900 repeats) in the first intron of the gene *FXN* (Campuzano et al., 1996; Monrós et al., 1997). The remaining patients (approximately 4%) are compound heterozygous with the expanded GAA repeat on one allele and another pathogenic variant (point mutation, deletion or insertion) on the other allele (Galea et al., 2016). The pathological GAA expansion affects the expression of *FXN* by blocking the transcription of mRNA through the formation of sticky DNA triplexes and R-loop structures (reviewed in Kumari and Usdin, 2012) and/or inducing abnormal heterochromatinization (Saveliev et al., 2003). Thus, the level of frataxin is strongly reduced from 5 to 30% of the physiological level (Campuzano et al., 1997). This reduction is related to the disease severity and length of the GAA repeat. In general, longer expansions are present in patients with a more severe phenotype such as earlier onset, faster progression and/or presence of non-neurological features (Parkinson et al., 2013). Although most of the pathogenic point mutations also decrease the level of functional protein, some lead to the production of a less active protein (De Castro et al., 2000; Bridwell-Rabb et al., 2011).

### Protein Frataxin

Frataxin is a small acidic protein, synthetized in the cytoplasm as a 210-residue polypeptide that is then imported into the mitochondria, where it is proteolytically processed to the mature and most abundant form of 130 residues [frataxin (81–210)] (Condò et al., 2007). Although frataxin has historically been considered a protein exclusively confined to the mitochondrial matrix, several studies have reported the existence of an extramitochondrial pool of frataxin in different human cell types (Acquaviva et al., 2005; Condò et al., 2006; Lu and Cortopassi, 2007). Extramitochondrial frataxin corresponds to the (81–210) mature form of the protein (Condò et al., 2010) and earlier studies suggested that after the initial transport and processing in the mitochondria, mature frataxin was transported back to the cytosol (Acquaviva et al., 2005; Condò et al., 2006). However, a recent study describes an N-terminal acetylated extramitochondrial form of frataxin in erythrocytes. This (76–210) isoform does not contain the mitochondrial targeting sequence, and remains in the cytosol where it is cleaved to produce an (81–210) protein identical to the mitochondrial mature form (Guo et al., 2018).

The three-dimensional structure of frataxin shows seven antiparallel β-sheets flanked by two α-helices, producing a characteristic globular αβ fold (Dhe-Paganon et al., 2000). The protein is ubiquitously expressed (Campuzano et al., 1996, 1997), but different cells types show distinct susceptibility to its deficiency. This could be explained by frataxin reduction in different tissues being associated with distinct transcriptomic profiles, as described recently (Chandran et al., 2017). Frataxin is a highly conserved protein through evolution, and its cellular function is critical for life in multicellular organisms. A strong reduction of frataxin in *Drosophila melanogaster* seriously affects viability (Anderson et al., 2005; Llorens et al., 2007). Knockout of the frataxin gene causes embryonic lethality in mice (Cossée et al., 2000) and in the plant *Arabidopsis thaliana* (Vazzola et al., 2007). In line with this, FRDA patients with non-GAA-repeat mutations in both frataxin alleles, resulting in totally deficient frataxin function, have not been reported.

At the time of *FXN* identification, there was no evidence about the function of frataxin. Experiments in yeast *Saccharomyces cerevisiae* promptly suggested a potential role for frataxin in iron homeostasis regulation. Deletion of the ortholog of frataxin in yeast (*Yfh1*) causes iron accumulation in the mitochondria (Babcock et al., 1997; Foury and Cazzalini, 1997) that was initially suggested at the expense of the cytosolic iron pool (Knight et al., 1998). However, measurements of cytosolic iron in frataxin-deficient yeast cells show that the cytosolic iron levels are not affected by the reduction of the frataxin synthesis (Li et al., 2012). Also associated with frataxin deficiency are reduced activities of mitochondrial iron-sulfur cluster (ISC) enzymes and aconitase, mitochondrial dysfunction and oxidative stress (reviewed in Calabrese et al., 2005; Lupoli et al., 2018). Since then, efforts have been made to answer several questions about iron in FRDA, including whether its accumulation is a primary or secondary event in the disease, what is the mechanism causing the deregulation and buildup of iron, and the role of this metal in the neuropathology of FRDA. Here, we review the contribution of the cellular and animal models of this disease and relevance of the studies using patient samples to gain knowledge about these issues.

## Iron Accumulation in Frda

Iron accumulation is a hallmark in FRDA and initially was suggested to be a primary pathogenic event triggered by frataxin deficiency. Tissues in which iron overload has been reported in FRDA patients are showed in Supplementary Table S1. The first observations linking iron and FRDA were made in postmortem heart samples from a few patients, where dense intracytoplasmic deposits of iron particles were detected in the cardiac fibers (Sanchez-Casis et al., 1976; Lamarche et al., 1980). These findings were later corroborated in subsequent studies that use different techniques for iron quantitation, such as Perls Prussian blue staining for histological detection or atomic absorption spectroscopy to measure total iron levels in tissue. The excess iron in the heart of FRDA patients appears as multifocal aggregates highly localized rather than as a diffuse pattern (Ramirez et al., 2012; Koeppen et al., 2015). This regional distribution of the excess of iron could be responsible for the findings of iron levels equivalent to healthy controls in bulk extracts or from random biopsied cardiac samples (Michael et al., 2006; Koeppen et al., 2015; Kruger et al., 2016). In addition to the heart, positive iron staining was observed in the liver and spleen of patients, showing a distribution consistent with a mitochondrial location (Bradley et al., 2000) as has been described in the frataxin-deficient yeast model (Babcock et al., 1997; Foury and Cazzalini, 1997).

In regions of the NS, such as DN and DRG, several scenarios regarding the iron content in FRDA have been reported: normal levels (Bradley et al., 2000; Koeppen et al., 2007, 2013; Solbach et al., 2014), accumulation (Bonilha da Silva et al., 2014; Harding et al., 2016), and limited and regional increases of this metal (Koeppen et al., 2009). These diverse results could be partly due to the technique used for iron measurements and their capability to discriminate between the accumulation and redistribution of iron. In most of these studies, the iron content has been quantified from a small number of patient samples, a situation that could also contribute to the different results reported. A more recent study with a higher number of FRDA subjects analyzed used magnetic resonance imaging, which allows *in vivo* iron quantitation (Harding et al., 2016). The authors found a significant increase in the iron concentration in the FRDA cohort compared with that in controls, in the DN and Red nucleus in the midbrain. However, the technical approach used in this study did not allow the authors to discriminate between deposition and redistribution or whether the location of the iron was extracellular or intracellular. Changes in the distribution of iron and other metals, such as copper and zinc, in the DN of FRDA patients have also been reported (Koeppen et al., 2012), although the total amount of these metals was similar to that of controls. It is worth highlighting that copper can also generate oxygen radicals and, in the context of FRDA, may exacerbate the oxidative injury caused by the increased amount of iron. Mitochondrial iron accumulation has also been documented in fibroblasts and lymphoblasts of FRDA patients. Although some authors have found an increase in the total iron content (Delatycki et al., 1999) or labile iron fraction (Tan et al., 2001), normal levels of iron have been reported in other studies (Wong et al., 1999; Sturm et al., 2005).

Several mouse models of FRDA have been obtained using different experimental strategies, and most of them show iron overload because of frataxin reduction (Supplementary Table S2). However, no iron accumulation was detected in embryonic samples of the frataxin knockout (KO) mice (Cossée et al., 2000), and the conditional model of frataxin deficiency in striated muscle (MCK-KO mice) showed heart intramitochondrial iron buildup only in late stages of the disease (Puccio et al., 2001). This led to the hypothesis that the increase in iron is not the causative pathological mechanism in FRDA and is a secondary effect.

Iron accumulation has been studied, particularly in muscle creatine kinase (MCK) conditional *Fxn* knockout mice (MCK-KO). This mutant develops a severe cardiac phenotype associated with almost ablation of frataxin in heart and skeletal muscle (Puccio et al., 2001). Different studies on this mouse line have reported distinct ages of onset for the iron overload despite using the same iron detection methodology (Supplementary Table S2). Some authors (Puccio et al., 2001) found iron deposits in 10-week-old mice, whereas animals 7 weeks of age did not present iron accumulation but showed deficits in ISC enzyme activities. By contrast, other authors (Huang et al., 2013) observed progressive iron accumulation from 5 to 10 weeks of age, with the presence of the iron deposits being the first observable histological change in the MCK-KO hearts. Therefore, there is no consensus about when iron starts to accumulate and whether the presence of iron deposits is relevant for the development of heart pathology in FRDA.

An interesting result found in the MCK-KO model is that iron levels increase in other tissues besides the heart (Supplementary Table S2), such as the liver, kidney and spleen, as well as serum (Huang et al., 2009; Whitnall et al., 2012). Because the amount of frataxin in these non-muscular tissues is within the normal range in the MCK-KO model (Huang et al., 2009; Whitnall et al., 2012), the iron increase was explained as an alteration of systemic iron metabolism orchestrated by the frataxin reduction in heart and skeletal muscle. It could be operated through a signaling mechanism involving hepcidin (Huang et al., 2009; Whitnall et al., 2012), a key regulator of systemic iron metabolism in mammals. These explanations indicate that excess iron plays a significant role in the disease. The mitochondrial iron in the heart of MCK-KO mutants is in the form of biomineral iron, which aggregates with phosphorus and sulfur (Whitnall et al., 2012). This is important because excess of iron that is not bound to ferritin, the main cellular protein that stores iron, can be very toxic by generating reactive oxygen species (ROS).

In the liver of mice with hepatic Fxn deficiency (Fxn^Alb^ mice), a fivefold increase in the mitochondrial iron level has been observed at 28 days of age compared with that in control mice. Meanwhile, 14-day-old mutant showed deficits in ISC enzymes in the liver but normal levels of iron (Martelli et al., 2015), suggesting that iron accumulation is a secondary event also in the liver. As in FRDA patients, iron deposition in the NS of FRDA mouse models has not been clarified yet. In conditional knockout models with frataxin reduction in neurons and other tissues (Puccio et al., 2001) or in parvalbumin-positive cells in the DRG, cerebellum and brain (Piguet et al., 2018), no iron accumulation has been observed. Other studies have reported few iron deposits in NS late in disease progression (Simon et al., 2004) or that iron accumulates both intracellularly and extracellularly in the brain following the loss of Fxn (Chen et al., 2016a). To understand these diverse findings, the authors have indicated that the different sensitivities of the techniques used to measure iron could be an explanation, or that, in some mouse models, cells loss may occur before sufficient iron is accumulated to be detected.

Other animal models of FRDA have also shown an increase in iron levels in the mitochondria (Supplementary Table S2), as is the case of *D. melanogaster* (Soriano et al., 2013). In this species, severe loss of frataxin function results in iron accumulation in multiple tissues, including the brain (Chen et al., 2016b). This was the first FRDA model clearly demonstrating that iron can accumulate in the NS. Additionally, frataxin-deficient flies also showed increased levels of other transition metals, such as zinc, copper and manganese, suggesting that frataxin reduction affects the homeostasis of other metals besides iron (Soriano et al., 2016). In the plant *A. thaliana*, frataxin is localized in both the mitochondria and chloroplasts, where it is suggested to have similar functions; its deficiency alters the iron content, and this metal accumulates in the two organelles (Martin et al., 2009; Turowski et al., 2015). These results indicate that frataxin-defective organisms show a common propensity to accumulate Fe into organelles where this protein is located. Although many studies have suggested that iron deposits are not instrumental and represent a late event in the pathogenesis of FRDA, the role of Fe in the disease pathophysiology is not fully understood. Recently, frataxin function has been nicely discussed (Alsina et al., 2018), and it can offer some insight into the mechanisms underlying iron accumulation in FRDA tissues and in model organisms. We review this subject in the following section.

## Frataxin Function and Potential Mechanisms of Mitochondrial Iron Accumulation

Iron is vital for the cell because it participates in essential biological processes such as DNA synthesis, gene regulation, cellular respiration, and energy production. The mitochondria play a major role in the cellular metabolism of this element and synthesis of heme and ISCs, which are needed for the function of many proteins involved in such cellular processes (Napier et al., 2005; Richardson et al., 2010). Iron homeostasis must be highly controlled because the depletion of its level can produce cellular death, and an increased level of iron promotes the production of ROS. In its Fe^+2^ state, iron can interact with oxygen via the Fenton and Haber-Weiss reactions to catalyze the production of ROS, which oxidize and damage proteins, lipids and nucleic acids (Eaton and Qian, 2002). Therefore, precise regulatory systems are needed to ensure the proper amount and oxidative state of iron in the cell and its different compartments.

Although frataxin has been proven to be a mitochondrial iron-binding protein (Foury et al., 2007) involved in the homeostasis of iron, its role remains unclear. Many studies have indicated that this protein could play roles related to iron storage (Park et al., 2003; Huang et al., 2011), heme (Yoon and Cowan, 2004; Napier et al., 2005) and ISC synthesis (Foury et al., 2007; Richardson et al., 2010; Lane et al., 2015) and could function as an iron sensor (Adinolfi et al., 2009) and a metabolic switch (Becker et al., 2002). Some of these possible roles have been more thoroughly tested and proven that others, with the involvement in ISC synthesis being the most accepted one thus far. We discuss these functions next and its possible relationship with mitochondrial iron accumulation in FRDA.

### Iron Storage

The observation that yeast frataxin (Yfh1), under aerobic conditions and an excess of iron, can form spheroidal oligomers that have ferroxidase activity (Adamec et al., 2000; Gakh et al., 2002; Park et al., 2002) promptly suggested that frataxin could function as an iron storage protein, similar to ferritin (Pandolfo and Pastore, 2009). These Yfh1 multimers have the capacity to bind up to 50 atoms of iron per monomer and maintain it in a redox-inactive state through the ferroxidase activity, which converts redox-active Fe^+2^ to Fe^+3^ (Gakh et al., 2006). On this line, frataxin could store iron in a bioavailable form in the mitochondria, thus its deficit would lead to iron loading in this organelle.

Frataxins are proteins capable of binding iron. Nuclear magnetic resonance (NMR) studies predominantly mapped the iron-binding sites in the conserved acidic α1 and β1 regions, which contain a clustering of negatively charged aspartate and glutamate residues (reviewed in Bencze et al., 2006; Pandolfo and Pastore, 2009). However, the hypothesis of frataxin as an iron storage protein presents several problems. Perhaps, one of most serious questions is that, in the case of human frataxin (FXN), the mature form of the protein is not prone to oligomerization. Only the precursor and intermediate forms of FXN can form oligomers and in an iron-independent manner (Adinolfi et al., 2002; O’Neill et al., 2005). Nevertheless, it has lately reported that the mature form of FXN, under specific experimental conditions can form oligomers, but they are unstable (Ahlgren et al., 2017). Other important caveat to this hypothesis is the redundant role that FXN would play with mitochondrial ferritin. In addition, it has been reported that oligomerization-deficient mutant Yfh1 is able to substitute functionally wild-type Yfh1 (Aloria et al., 2004), indicating that oligomerization is not required for frataxin function.

### Heme Biosynthesis

Frataxin can bind iron directly without forming oligomers (Bencze et al., 2006; Cook et al., 2006). Depending on the organism (bacteria, yeast or human) and state of iron (Fe^+2^ or Fe^+3^), different possible iron-binding sites in the protein have been described (Yoon and Cowan, 2003; Bou-Abdallah et al., 2004; Cook et al., 2006; Yoon et al., 2007; Huang et al., 2008). The primary site that binds Fe^+2^ involves the residues 18, 19 and 22 of CyaY (and corresponding ones in Yfh1 and FXN) at the acidic ridge in the α1 helix of frataxin. This raised the possibility of new functions for frataxin in iron-dependent processes, in which some of the involved machinery components seem to interact with FXN. Frataxin interactions have been reported with mitochondrial aconitase, ferrochelatase (Fech), succinate dehydrogenase and the components of the ISC synthesis machinery (Gerber et al., 2003; Yoon and Cowan, 2003; Bulteau et al., 2004; Bencze et al., 2006). Among these interactions, the binding of frataxin with components of the ISC complex has been reproduced and characterized in independent studies (reviewed in Martelli and Puccio, 2014).

The heme biosynthetic pathway is carried out in mitochondria. This pathway includes several enzymes (Sassa, 2006; Furuyama et al., 2007; Chung et al., 2012; Dailey and Meissner, 2013; Chiabrando et al., 2014), with the 5-aminolevulinic acid synthase (ALAS) being the first one in that pathway and the rate-limiting enzyme. ALAS has two isoenzymes, ALAS1 and ALAS2. Whereas ALAS1 expression is negatively regulated by heme directly bound to a cysteine-proline dipeptide motif (Furuyama et al., 2007), the ALAS2 gene contains an iron-responsive element (IRE) that interacts with iron regulatory proteins (IRPs) regulating its expression at the posttranscriptional level. Under iron deficiency conditions, the binding of IRP to ALAS2 mRNA inhibits its translation; by contrast, under iron sufficiency conditions, IRPs detach from the IRE, resulting in increased ALAS2 translation. In this manner, the cytosolic iron availability regulates the rate of heme synthesis (Ponka, 1997).

The final enzyme in the heme biosynthetic pathway is Fech, which inserts ferrous iron into protoporphyrin IX. This enzyme contains a [2Fe-2S] cluster that is necessary for enzymatic activity (Wu et al., 2001). The disruption in ISC synthesis could adversely affect the rate of heme synthesis (Richardson et al., 2010; Huang et al., 2011). It has been shown that mitoferrin (Mfrn1) is responsible for iron transport into mitochondria, allowing the direct transfer of ferrous iron for heme and/or ISC synthesis (Fleming, 2011). The role of frataxin in heme synthesis is not clear, although a reduction of heme synthesis was observed in yeast and mouse frataxin mutants (Lesuisse et al., 2003; Huang et al., 2009). Different studies have suggested a direct role of frataxin in the heme synthesis pathway by binding directly to Fech and acting as an iron donor (Yoon and Cowan, 2004; Napier et al., 2005). Thus, this metal would accumulate because of frataxin deficiency.

### ISC Biogenesis Chaperone

Iron-sulfur clusters are protein cofactors present in almost all living organisms. They play a critical role in several cellular functions, from electron transport in mitochondrial respiratory complexes to DNA repair or metabolism. The *de novo* synthesis of ISC occurs in the mitochondria (Lill, 2009; Beilschmidt and Puccio, 2014). The components of the ISC synthesis machinery are the cysteine desulfurase (NFS1), which converts L-cysteine to L-alanine with the generation of sulfur, an accessory protein (ISD11), a reductant (ferredoxin), a scaffold protein (ISCU) and an iron delivery protein that is at the core of the debate (Lill and Mühlenhoff, 2006; Py and Barras, 2010). Besides ISD11, recent findings showed that human NFS1 needs another accessory protein, the mitochondrial acyl carrier protein (ACP), making the cysteine desulfurase complex (NFS1-ISD11-ACP) (Van Vranken et al., 2016). In addition, NFS1 requires FXN for its full function (Tsai and Barondeau, 2010; Fox et al., 2015). Regarding the role of FXN in the ISC synthesis, there are two main hypothesis. It was first suggested that frataxin could be the iron donor (Gerber et al., 2003; Yoon and Cowan, 2003; Layer et al., 2006). Using a combination of different techniques based on isothermal titration calorimetry and multinuclear NMR spectroscopy, Cai et al. (2018) have recently showed that FXN can function as an iron donor *in vitro*. They found that FXN-Fe^2+^ interacts weakly to the NFS1-ISD11-ACP complex, but the presence of ISCU largely improves this interaction and that dissociation of Fe^2+^ from FXN occurs when the L-cysteine and ferredoxin enters into the equation. These results suggest that FXN is the proximal source of iron for ISC assembly, but *in vivo* characterization is required to confirm this role for frataxin.

Other studies have proposed that frataxin could actually act as a regulator in the synthesis of ISC (Adinolfi et al., 2009; Tsai and Barondeau, 2010; Bridwell-Rabb et al., 2012). The binding of frataxin to the ISCU/NFS1/ISD11 complex can stabilize it, activates the cysteine desulfurase activity and control the entry of iron to the complex (Tsai and Barondeau, 2010; Colin et al., 2013; Pandey et al., 2013).

Mutation of specific aspartate and glutamate residues in the acidic ridge of frataxin, where iron-binding sites were previously mapped, alters the interaction of frataxin with ISCU. Unexpectedly, these mutations does not reduce the total number of iron-binding sites (Schmucker et al., 2011). These results suggested that some of these residues might be critical for frataxin interaction with the core components of the ISC complex, but not for binding iron. Consequently, some authors (Gentry et al., 2013) proposed that frataxin might have unknown binding sites for this metal. Using different spectroscopic and structural methodologies, a new iron-binding site was identified at His86 in the N-terminus of the mature FXN form, therefore not positioned in the acidic ridge. Interestingly, this residue was essential for iron binding and for *in vitro* productive synthesis of ISC by ISCU, without showing a direct interaction with the scaffold protein (Gentry et al., 2013). These results support the proposal that frataxin is the iron donor for ISC assembly.

Extramitochondrial frataxin has been shown to physically interact with IscU1, the cytosolic isoform of the human ISC, indicating a role for frataxin in the Fe/S cluster assembly in the cytosol (Acquaviva et al., 2005). The cytosolic form of frataxin has also been reported to interact and possibly regulate the function of the cytosolic aconitase/iron regulatory protein-1 (IRP1) (Condò et al., 2010). Other functions suggested for frataxin in the cytosol include preventing oxidative stress and apoptosis (Condò et al., 2006); and in the nucleus, frataxin could promote DNA repair and telomere stability (Lill et al., 2015).

### Iron Sensor and Metabolic Switch

As indicate above, frataxin could act as an iron sensor to tightly regulate ISC synthesis (Adinolfi et al., 2009). In *E. coli*, CyaY, far from facilitating iron delivery, can inhibit the ISC synthesis process. With normal iron levels, CyaY has a low affinity for the IscS-IscU system (the bacterial ISC synthesis machinery), and ISC is loaded to the final acceptor. However, under conditions of iron excess, the affinity of CyaY for IscS would increase, slowing the rate of ISC synthesis and avoiding an overproduction of ISC. This would avoid the degradation of ISC not inserted into an acceptor protein and the release of free iron that could catalyze the production of ROS. However, the consequence of frataxin deficiency in FRDA patients is a decrease in ISC protein function, suggesting that FXN might operate as an activator of the ISC synthesis. *In vitro* studies also support the role of FXN as an allosteric activator of the ISC assembly (Tsai and Barondeau, 2010). In contrast, both FXN and CyaY can complement a yeast strain with deletion of *Yfh1*, indicating that eukaryotic and bacterial frataxins can at least partially conserve their functions (Cavadini et al., 2000; Bedekovics et al., 2007). To clarify this controversial results, Bridwell-Rabb et al. (2012) conducted enzyme kinetic assays in which analogous components of the human and *E. coli* ISC complex were interchanged. Interestingly, they found that CyaY could substitute FXN as an activator of the cysteine desulfurase in the human ISC complex. Conversely, FXN could also replace CyaY as an inhibitor in the assembly of the bacterial ISC. Therefore, both frataxin proteins have the property to modulate the ISC formation, but the mechanism of action of any of them, as an activator or inhibitor of ISC biogenesis, depends on another component of the ISC assembly machinery. This component was found to be the cysteine desulfurase, which controls how the frataxin protein operates in the ISC complex (Bridwell-Rabb et al., 2012). Differences between the human and bacteria cysteine desulfurase might explain the different role of frataxin in humans with respect to *E. coli* and by extension in eukaryotes vs prokaryotes. Further studies are needed to define the nature of these differences that dictate how frataxin acts in the process of the ISC synthesis.

Examination of the role of frataxin in ISC and heme metabolism in differentiating erythroid cells has suggested that frataxin acts as a metabolic switch in the diversion of iron from one metabolic pathway to another. This was supported by the observation that an increased level of protoporphyrin IX (PPIX) leads to the downregulation of frataxin expression and potential diversion of iron from ISC synthesis toward heme synthesis (Becker et al., 2002). The molar ratio between frataxin and Fech affects the rate of heme synthesis (Yoon and Cowan, 2004), and these findings have suggested that frataxin could function as a metabolic switch in regulating mitochondrial iron metabolism, independently of the expression levels of PPIX or Fech. This metabolic switch allows the mitochondria to favor heme or ISC syntheses, depending on the frataxin levels. The possible mechanism behind this switching may be the relative affinities of frataxin for Fech vs ISCU. This may result in the preferential binding of frataxin to Fech over ISCU at relatively lower frataxin levels (Richardson et al., 2010).

When the frataxin level is reduced, there would be no control in the rate of ISC formation, causing iron accumulation (Adinolfi et al., 2009). Regarding the metabolic switch role of frataxin, its depletion would provoke the enhancement in the reduction of ISC formation at the ISCU machinery level and at the end, subjecting the cell to increased iron accumulation and ROS production (Yoon and Cowan, 2004).

### Dysregulation of Iron Homeostasis in FRDA

**FIGURE 1:**
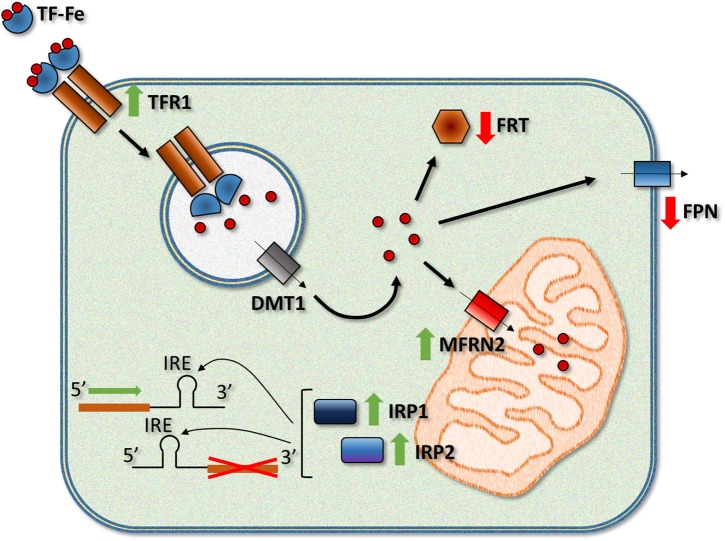
Frataxin deficiency leads to a series of alterations in the activity and/or expression of different iron-related proteins. Among the most relevant changes observed in humans and FRDA models, there is an increase in the activity of the iron regulatory proteins IRP1 and IRP2, upregulation of TFR1 (transferrin receptor 1) and the mitochondrial iron importer MFRN2 (mitoferrin-2), and downregulation of the iron exporter FPN (ferroportin) and iron storage proteins FRTs (ferritins, light and heavy chains). TF, transferrin; DMT1, divalent metal transporter 1.

Although the mechanisms responsible for mitochondrial iron loading in FRDA are not fully understood, there are several reasonable hypotheses that could explain this alteration. In the MCK-KO mouse, Huang et al. reported altered heme and ISC synthesis and mitochondrial iron storage. Significantly diminished activity and expression of ISC enzymes, such as NFS1 and ISCU1/2, were observed that agreed with the reduction in the expression of succinate dehydrogenase complex, subunit A (Sdha) (Huang et al., 2009). At the same time, other studies have shown a downregulation in the number of key heme synthesis enzymes and decreased heme levels in the heart of the MCK KO model (Schoenfeld et al., 2005; Huang et al., 2009). A decrease in the synthesis of different products that require iron in the mitochondria could explain the accumulation of this metal because it cannot be exported from the organelle being part of those products. However, frataxin deficiency can also affect the expression of several other proteins, some of them critical for the proper control of iron metabolism (Figure 1). In DN, Koeppen et al. (2007) observed modification of the expression of proteins such as transferrin receptor 1 (TFR1), ferritins (FRTs) and ferroportin (FPN), alterations shared among animal models of FRDA. In the MCK-KO mouse (Martelli and Puccio, 2014), has been described marked TFR1 upregulation, leading to increased iron uptake, and the downregulation of FPN, decreasing cellular iron release from the cytosol. There are also decreased levels of ferritin light chain 1 (FRTL1) and heavy chain 1 (FRTH), implying reduced cytosolic iron incorporation and storage. Finally, an increased expression of Sec15l1 (an exocyst complex protein) and the mitochondrial iron importer mitoferrin-2 (MFRN2) in the mutant could facilitate mitochondrial iron influx, which was seen to be enhanced in this model (Whitnall et al., 2008).

The link between frataxin deficiency and alteration in the expression of some of these proteins could be IRP1 and IRP2. Both proteins bind to IRE elements in the 5′ and 3′ UTR of the mRNAs of iron metabolism-related proteins (Chiang et al., 2016). When bound to a 5′ IRE, IRP1 and IRP2 repress the translation of the mRNAs while binding of the IRPs to a 3′ IRE increases the stability and translation of the mRNAs. The activity of both IRPs is increased by the low levels of cellular iron by different mechanisms. Interestingly, IRP1 activity is controlled by the insertion of an ISC, which converts the protein into a cytosolic aconitase and makes it unable to bind to IRE elements. Since frataxin depletion reduces ISC synthesis, it is reasonable to suggest a role for IRP1 in the deregulation of iron metabolism. Nevertheless, the activity of IRP2, which is controlled by iron-dependent controlled ubiquitination and degradation, is also increased in MCK-KO mouse, possibly due to cytosolic iron depletion (Whitnall et al., 2008). More recently, Martelli et al. (2015) studied a double KO mouse with complete ablation of *Irp1* and liver-specific deletion of *Fxn*. In these mice, the authors observed that alterations in the expression of proteins such as TFR1 and divalent metal transporter 1 (DMT1) were indeed due, a least partially, to IRP1, while the regulatory protein seemed to exert little effect on the expression of other proteins such as FRTL and FPN. Other models of the disease also manifest similar alterations in the expression of proteins involved in iron metabolism. Frataxin depletion in *D. melanogaster* produces alterations in the expression of mitoferrin, IRP-1A and ferritin, while genetic modifiers for *Malvolio*, *Tsf1* and *Tsf3* (*Drosophila* homologs of DMT1 and transferrins) and *IRP-1A* and *IRP-1B* could correct the motor deficiency phenotype of frataxin-deficient flies (Soriano et al., 2016; Calap-Quintana et al., 2018; Monnier et al., 2018). Although some hints point to the possible causes of iron dysregulation in FRDA, more research is needed to propose a more precise and final hypothesis.

## Mechanisms of Iron Neurotoxicity in Frda

**FIGURE 2:**
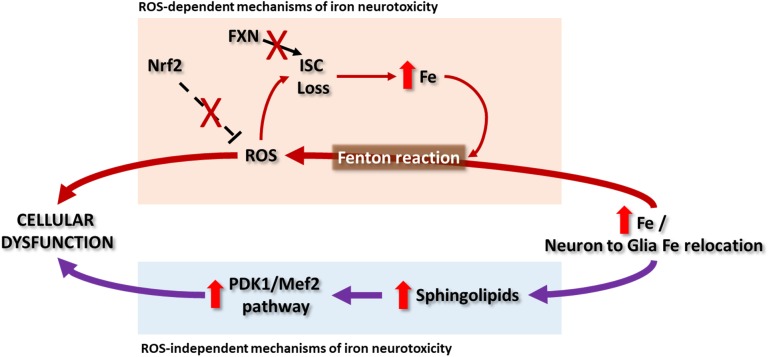
In Friedreich ataxia patients, deposits of iron in NS have not been clearly reported and it remains a controversial topic, while some evidences point to a relocation of iron from neurons to glial cells. So far there are some hypotheses that explain the possible mechanisms of iron toxicity that lead to cellular dysfunction. The ROS-dependent hypothesis proposes the iron-mediated catalysis of ROS by Fenton reaction. Besides the impairment in ISC synthesis due to frataxin deficiency, the radical species can in turn damage the ISC of proteins generating new free iron that will participate in the Fenton reaction. Contributing to this cycle is the observation that the Nrf2 signaling is defective in some cells and models of the disease. Another recent hypothesis, independent of ROS production, was explored in *Drosophila* and mouse models. It suggests that the cellular dysfunction can be due to the activation of the PDK1/Mef2 (3-phosphoinositide dependent protein kinase-1/myocyte enhancer factor-2) pathway by means of an increase in sphingolipids synthesis. Both hypothesis as well as the relevance of ROS in the pathology of FRDA have partial support, since the evidence pointing to them vary depending on the study.

Initially, it was postulated that iron overload in FRDA activates the formation of ROS via the Fenton reaction, which would arbitrate the pathogenesis of the disease. The highly redox active environment in the mitochondria could promote ROS generation when the free iron content increases. Nevertheless, an ROS independent mechanism mediated by iron has recently been proposed for FRDA neurodegeneration. These two mechanisms are described in the following paragraphs (Figure 2).

### ROS-Dependent Mechanism of Iron Neurotoxicity

Iron accumulation in the NS is a common feature in neurodegenerative diseases, including Alzheimer’s disease (Connor et al., 1992), Parkinson’s disease (Gorell et al., 1995), Huntington’s disease (Dumas et al., 2012) or amyotrophic lateral sclerosis (Kwan et al., 2012). In these disorders, iron deposits are observed in specific regions of the NS. As indicated previously, in Friedreich ataxia patients, deposits of iron have not been clearly reported in NS, and the increment of iron levels in the DN remains controversial (Waldvogel et al., 1999; Koeppen et al., 2007; Harding et al., 2016). This region is interesting because the patients develop neuronal atrophy and abnormal proliferation of the synaptic terminal, called grumose degeneration. The Koeppen’s group confirmed the presence of iron metabolism alteration in the NS of FRDA patients by changes in the expression of proteins related to iron, such as FPN, FRT and TFR1, in the DN (Koeppen et al., 2012) and DRG (Koeppen et al., 2009). They observed a relocation of iron from the affected neurons to the microglia, in the case of DN, or from neurons to the satellite cells in the DRGs. The increment of iron levels correlates with the production of ROS in these areas, suggesting that alterations of iron homeostasis could contribute to the pathogenesis of the disease through ROS production. Additionally, frataxin deficiency is related to low antioxidant defenses (Chantrel-Groussard et al., 2001; Paupe et al., 2009), making the nervous cells have an oxidative stress-prone phenotype.

The toxicity of iron may be due to its localization, biochemical form or cell ability to prevent the generation and propagation of iron. In living organisms, iron can be found in its reduced form, ferrous iron (Fe^2+^), and its oxidized form, ferric iron (Fe^3+^). Most iron binds, as a prosthetic group, to proteins such as hemoglobin, myoglobin, cytochromes or storage proteins; only less than 5% of iron in the cell is in the labile iron pool (LIP) (Li and Reichmann, 2016). The cellular LIP is defined as a pool of chelatable and redox-active iron complexes (Kakhlon and Cabantchik, 2002). LIP has the capacity to participate in the formation of harmful free radicals including the hydroxyl radical. Fe^2+^ is catalyzed to Fe^3+^ in a chemical reaction mediated by hydrogen peroxide (H_2_O_2_), which leads to the formation of hydroxide radical (OH∙). Cytotoxic events are started in the cell via the Fenton reaction. To avoid the negative effects of excess free iron, it is coupled to proteins such as FRT, which is responsible for iron storage inside the cell. In the satellite cells of DRG from FRDA patients, an increment of cytosolic FRT (Koeppen et al., 2009) was observed; in DN, ferritin is expressed mostly in oligodendrocytes, satellite cells around the neurons. As the disease progresses, the neurons begin to undergo atrophy, and these cells disappear, producing microglia FRT-positive cells (Koeppen et al., 2007). Noteworthy, mitochondrial FRT has not been detected in DRG neurons, a protein responsible to store free mitochondrial iron and with a very important role in antioxidant activity (Koeppen et al., 2007; Campanella et al., 2009). FPN is a transmembrane protein that exports iron and is expressed in the central NS and DRG. Interestingly, in the DRG from FRDA patients, the expression of FPN is exclusive to satellite cells (Koeppen et al., 2009), probably as a response to the iron release from DRG neurons to these cells (Koeppen et al., 2016). In the synaptic terminals of neurons from the DN of FRDA patients, is observed an increment of the ferroportin levels (Koeppen et al., 2007). The nerve endings present an elevated number of mitochondria, suggesting a response in the biosynthesis of this protein due to the dyshomeostasis of iron. Thus, the toxicity of this metal could be an important factor in grumose degeneration.

Because mitochondria are an important generator of ROS, the continuous presence of iron in the mitochondrial matrix makes it susceptible to new production of ROS. Proteins with ISC clusters, such as complex I, II and III of the electron transport chain, can be damaged by ROS. These complexes show reduced activity in FRDA patients (Rötig et al., 1997), probably because of iron toxicity. Moreover, these proteins will generate new free iron that will participate in the Fenton reaction and carry out new oxidative processes. In plasma and urine from FRDA patients (Emond et al., 2000; Schulz et al., 2000), similar to in all the disease models generated (González-Cabo and Palau, 2013), oxidative stress markers have been detected.

In the last years, neuronal models of the disease have been developed to better understand the neurodegenerative process. Oxidative stress, mitochondrial dysfunction, and iron dyshomeostasis are features shared among the neuronal models. For example, the granulate cells of the cerebellum from YG8R mice show an increment of ROS, lipid peroxidation and reduced glutathione levels (Bradshaw et al., 2016); the neurons of DRG from YG8R mice present an increment of ROS, dysfunctional mitochondria, and calcium dyshomeostasis (Mollá et al., 2017); the motor neuron cell line (NSC34) with reduced expression of the frataxin gene shows an increment in the GSSG/GSH ratio and more production of ROS (Piermarini et al., 2016) and alteration in complex I activity of the electron transport chain (Carletti et al., 2014); reduction of *Drosophila* frataxin (fh) expression in glial cells produces massive accumulation of fatty acids and increased sensitivity to oxidative stress (Navarro et al., 2010).

Under oxidative stress, the transcription factor Nrf2 binds to antioxidant response element (ARE) and modulates the expression of many genes involved in the antioxidant defense. It has been reported that Nrf2 signaling is defective in fibroblasts from FRDA patients (Paupe et al., 2009), the DRG and cerebellum of YG8R mice (Shan et al., 2013) and motor neuron and Schwann cell models (D’Oria et al., 2013; Shan et al., 2013), making these cells and tissues more vulnerable to oxidative stress damage and the neurodegeneration process. Concretely, frataxin deficiency in neurons promotes axonal dystrophy with multiple axonal spheroid formation mainly due to Ca^2+^ imbalance and oxidative stress (Mollá et al., 2017). The axonal swellings are associated with specific cytoskeletal changes (Mincheva-Tasheva et al., 2014; Piermarini et al., 2016; Mollá et al., 2017), improper axonal transport and autophagic flux (Shidara and Hollenbeck, 2010; Mollá et al., 2017). Moreover, the oxidative stress damage effects to the neurites growth leads to shorter dendrites due to alterations in the dynamics of the microtubules (Shan et al., 2013; Carletti et al., 2014; Piermarini et al., 2016). When oxidative stress is reduced in these neurons, either by direct addition of antioxidants (Piermarini et al., 2016) or through drugs that increase the expression of Nrf2 (Petrillo et al., 2017), axonal growth is improved. In any case, these alterations affect the cellular viability of both neurons and glia, triggering cell death processes either by apoptosis (Mincheva-Tasheva et al., 2014; Igoillo-Esteve et al., 2015; Katsu-Jiménez et al., 2016) or autophagy (Bolinches-Amorós et al., 2014; Edenharter et al., 2018). It has been shown that iron is a key element in the induction of cell death (Dixon et al., 2012). This new process, called ferroptosis, is dependent on iron and is accompanied by an increase in ROS and lipid peroxidation and a decrease in GSH. As we have reviewed previously, these characteristics are observed in frataxin-deficient cells; therefore, new studies are needed to determine the possible cell death due to a ferroptosis process.

### ROS-Independent Mechanisms of Iron Neurotoxicity

The extent to which oxidative stress is necessary for the pathogenesis of FRDA is, however, controversial. The deficit in ISC enzymes and increase in iron levels were not paired with an increase in ROS in the MCK-KO mouse (Seznec et al., 2005), in Fxn^Alb^ mice with specific depletion of Fxn in liver (Martelli et al., 2015), and in the inducible and reversible FRDAkd mouse model (Chandran et al., 2017). Similarly, increased oxidative stress was not detected in several *Drosophila* knockdown models, and overexpression of ROS scavengers did not rescue the viability, heart dysfunction or neurodegeneration phenotypes (Anderson et al., 2005; Shidara and Hollenbeck, 2010; Tricoire et al., 2014; Chen et al., 2016b). Moreover, FRDA neural tissue did not show differences in the susceptibility to oxidative stress in a study using patient samples (Macevilly and Muller, 1997). The absence of ROS markers in these studies might be attributed to differences in methodology or the distinctive features of each of the animal models or tissues employed. Alternatively, it was suggested that the discrepancies in the presence of ROS would be explained by differences in the timepoints studied, in a scenario where oxidative stress would be a secondary effect rather than a primary cause, occurring later in the progression of FRDA (discussed in Lupoli et al., 2018). Recent findings in the fly and mouse models of FRDA actually support a mechanism that is independent of ROS that links neurodegeneration and iron accumulation via the sphingolipid pathway (Chen et al., 2016a,b).

Alterations in the homeostasis of lipids are not exclusive of FRDA (Huang et al., 1978; Filla et al., 1980; Thurman and Draper, 1989; Raman S.V. et al., 2011; Worth et al., 2014) and have been reported in neurodegenerative disorders, including Parkinson’s, Alzheimer’s, Huntington’s, multiple sclerosis and Niemann–Pick diseases (reviewed in Shamim et al., 2018). In the case of FRDA, lipid droplet accumulation was first described in the cardiac muscle of the NSE-KO mouse model (Puccio et al., 2001) and was further characterized as pathological in a glial frataxin knockdown model in *Drosophila* (Navarro et al., 2010). The accumulation and dyshomeostasis of lipids have been reported as well in several other cellular and animal models of the disease (Martelli et al., 2012; Schiavi et al., 2013; Obis et al., 2014). More recently, the expression of a mutant allele of the *Drosophila fh* in mosaic clones produced an age-dependent degeneration of photoreceptor neurons with impaired mitochondrial function, increased levels of Fe^2+^ and Fe^3+^ in multiple tissues and dramatic lipid droplet accumulation in glial cells (Chen et al., 2016b), consistent with the work from Navarro et al. (2010). Because the Fenton reaction can induce lipid peroxidation, oxidative stress has been classically proposed to be the link between abnormal lipid homeostasis and neurodegeneration in FRDA. However, Chen et al. did not observe an increase in free radical production and, neither providing the antioxidant AD4 nor overexpressing the ROS scavengers catalase, SOD1 and SOD2, suppressed neurodegeneration (Chen et al., 2016b). Moreover, reducing the number of lipid droplets by overexpressing brummer lipase or lipase 4 was insufficient to improve the neurodegeneration defect in the knockout photoreceptors. Because high iron conditions increase sphingolipid synthesis in wild-type yeast (Lee et al., 2012), Chen et al. (2016b) pursued the characterization of this pathway in their fly model. The synthesis of sphingolipids, including dihydrosphingosine, dihydroceramide and sphingosine, was actually increased in the *fh* mutants. Both the genetic knockdown and chemical inhibition of the fly serine palmitoyltransferase, an enzyme essential for the *de novo* synthesis of sphingolipids, rescued neurodegeneration. Additionally, they found that downstream targets of the transcription factor Mef2 were upregulated in response to the increase in sphingolipids in *fh* mutants and knocking down the Pdk1/Mef2 pathway led to the suppression of photoreceptor degeneration. To ask whether this novel pathway described in *Drosophila* was conserved in vertebrates, Chen et al. (2016a) developed a mouse model with reduced *Fxn* levels in the NS. Both ferrous and ferric iron were found to be increased in the cerebral cortex and, consistent with their observation in the fly model, they could not find increased ROS production measured as levels of lipid peroxidation. Loss of *Fxn* in the NS led to the activation of the PDK1/Mef2 pathway in the mouse before the appearance of the neurological manifestations. Sphingolipids and PDK1 activation were also found to be increased in cardiac tissue from FRDA patients (Chen et al., 2016a). Nevertheless, alterations in the PDK1/Mef2 pathway were not replicated in the FRDAkd-inducible mouse model of FRDA (Chandran et al., 2017). In this study, the authors did not find changes in PDK phosphorylation in the brain, muscle, heart or liver in the chronic adult knockdown mice. The levels in the cerebellum and heart of five target genes of *Mef2* were analyzed as well and displayed changes that were inconsistent across tissues. Alternatively, other biochemical pathways could be altered in relation to iron under frataxin-deficient conditions. In line, several genes involved in hemochromatosis, an iron-overload disorder, are upregulated in the FRDAkd-inducible mouse model, in which no increase in ROS and no strong evidence of Pdk1/Mef2 activation were observed (Chandran et al., 2017).

## Potential Treatment of Frda by Reducing Iron Toxicity

An optimal therapy in FRDA could consist of providing patients with sufficient frataxin amount to restore the functions of this protein. Several strategies are being developed to obtain this objective, such as gene therapy (Perdomini et al., 2014; Piguet et al., 2018), transplanting hematopoietic stem and progenitor cells (Rocca et al., 2017) or improving *FXN* transcription (Soragni and Gottesfeld, 2016). While advancing the development of such strategies for its application to patients, rational treatment could be considered to remove excess iron.

The utilization of iron chelators is one of the first strategies proposed to treat FRDA patients to eliminate excess iron accumulation in the mitochondria. To that end, an iron chelator should be able to cross the cell membranes, access mitochondria and redistribute the excess labile iron. Deferiprone (DFP, 1, 2 dimethyl-3-hydroxy-pyrid-4-one), an orally administered lipid soluble iron chelator can easily cross the blood–brain barrier and cellular membranes. DFP shows relatively low affinity for iron and can transfer this metal to circulating transferrin, as well as to cellular acceptors—e.g., for heme synthesis (Sohn et al., 2008). Therefore, its use is less probable to produce iron depletion than other known chelators. For all these reasons, DFP has been considered as the prototypic compound for iron redeployment (Cabantchik et al., 2013). It is also used to treat iron-overloaded patients with β-thalassemia who need continuous blood transfusions (Totadri et al., 2015), and their properties make it an interesting therapeutic compound for FRDA.

The use of DFP in cellular and animal models of FRDA has beneficial results, e.g., frataxin-deficient HEK-293 cells could restore mitochondrial dysfunction after DFP treatment. It was attributed to the ability of this compound to make available the iron accumulated into mitochondria (Kakhlon et al., 2008). These conclusions were also supported by our group using a *Drosophila* FRDA model, in which DFP improved the survival and climbing ability phenotypes because of the removal of excess free mitochondrial iron, thereby preventing its toxic effects (Soriano et al., 2013). Recently, it has been reported that DFP is effective to reduce ROS production and improve calcium handling kinetics in an FRDA human-induced pluripotent stem cell-derived cardiomyocyte model (Lee et al., 2016).

The efficacy of DFP has already been assessed in clinical trials in FRDA patients, but the results are inconclusive. However, considering these studies, DFP has shown beneficial effects on cardiac parameters achieved at low doses of the compound, while treatment with higher doses of DFP could aggravate the disorder by affecting the ataxia phenotype (Pandolfo et al., 2014). A high dose of DFP could remove too much iron, provoking a deficiency of this metal, which could affect the cellular functions that require iron. On the other hand, the positive action of this drug on the heart compared with NS has also been related to the initiation of treatment before cardiac dysfunction is present (Elincx-Benizri et al., 2016). Overall, DFP seems to have therapeutic value in FRDA and is quite safe at lower doses, but systematic, large scale, placebo controlled longer studies are needed to evaluate its usefulness (Strawser et al., 2017). In line with these issues, our results using a *Drosophila* model of the disease indicate that DFP treatments at the larval stage recover FRDA-like phenotypes in flies, but no improvement was observed when the drug is administered to adults (Soriano et al., 2013). Additionally, we found that reducing the excess of iron accumulating into mitochondria through a genetic strategy has benefit in this FRDA model (Navarro et al., 2015; Soriano et al., 2016). These results show a causal relationship between iron and frataxin deficiency. Thus, iron is a critical factor in the pathophysiology of FRDA and appears not be a simple epiphenomenal event in the disease. Consequently, research on how to deal with the iron excess and eradicate the iron toxicity is still an area of great interest.

In the last decades, some small molecules with pharmacophore characteristics for iron chelation are being explored for treatment in neurodegenerative diseases with regional increase of iron levels (reviewed in Singh et al., 2018). These compounds are derivatives of the 8-hydroxyquinoline, hydroxypyridone, and arylhydrazones, and some of them are able to reduce selectively the iron excess in the mitochondria (Mena et al., 2015). The use of pro-chelating compounds, which are activated in the target region, have the benefit to remove the excess of iron in the affected regions. Thus, this strategy would protect cells in non-affected areas from the risk of iron subtraction. Novel pro-chelators triggered by H_2_O_2_ were obtained as promising candidates for management of Parkinson’s disease (Perez et al., 2008). Similarly, this approach might be useful in FRDA since H_2_O_2_ is a critical pathogenic agent associated to frataxin deficiency (Anderson et al., 2008). Finally, new compounds with multifunctional actions, as previously indicated for DFP, are also being examined for effective treatment of neurodegenerative diseases (Singh et al., 2018). Several of these diseases, including FRDA, share some pathological features such as regional iron increase, oxidative stress and dysregulation of calcium signaling. Recently, a novel compound, named CT51 (N-(1,3-dihydroxy-2-(hydroxymethyl)propan-2-yl)-2-(7-hydroxy-2-oxo-2H-chromen-4 yl) acetamide), has been synthesized that seems to suppress these cellular impairments (García-Beltrán et al., 2017). CT51 can easily permeate the cell membrane and distribute to both mitochondria and the cytoplasm. It works as a highly selective iron chelator and possesses significant capability for free radical quenching, protecting cells against oxidative damage. The use of CT51 in primary hippocampal neurons decreases the continuous release of calcium provoked by an agonist of ryanodine receptor-calcium channels, supporting a protective role of this compound in neuronal function. Although developments of this kind of drugs are not a curative strategy, because the frataxin deficiency will persist, they might improve the current pharmacological treatments and might produce increases in quality of life and slowing of disease progression.

## Conclusion

The relationship between neurodegeneration and iron accumulation is well established. However, in Friedreich’s ataxia, it remains unclear whether iron loading is the cause or consequence of the pathophysiology. The studies in model organisms suggested both hypotheses in relation to the role of iron in this disease: a key role in the pathophysiologic mechanisms or a secondary event that appeared late in the disease progression. It was also debated the extent to which oxidative stress is crucial to explain the pathogenesis of FRDA. Indeed, additional pathways might be involved in the toxicity of iron independent of ROS production. Differences in the distinctive features of each of the FRDA models studied, vulnerability of the different tissues and cells to frataxin deficit and timepoints analyzed could partially explain the contradictory results. Nevertheless, from the therapeutic point of view, it is interesting to explore whether new iron chelators showing protective properties on cellular function could improve the mitochondrial dysfunction in FRDA.

## Author Contributions

JL and MM planned the manuscript. JL, SS, PC-Q, PG-C, and MM wrote the manuscript and agreed on the finally submitted version of the manuscript.

## Conflict of Interest Statement

The authors declare that the research was conducted in the absence of any commercial or financial relationships that could be construed as a potential conflict of interest.
