# Supplementary material for: The Role of Iron in Friedreich’s Ataxia: Insights From Studies in Human Tissues and Cellular and Animal Models
Source: Front Neurosci. 2019 Feb 18;13:75. doi: 10.3389/fnins.2019.00075 (PMC6387962; doi:10.3389/fnins.2019.00075)
Supplement: Supplementary file 1 [file Table_1.docx]

Supplementary Material

The role of iron in Friedreich’s ataxia: insights from studies in human tissues and cellular and animal models

**José Vicente Llorens^*^, Sirena Soriano, Pablo Calap-Quintana, Pilar González-Cabo, María Dolores Moltó^*^**

*** Correspondence:** Dr. José Vicente Llorens, [j.vicente.llorens@uv.es](mailto:j.vicente.llorens@uv.es); Dr. María Dolores Moltó, [dmolto@uv.es](mailto:dmolto@uv.es)

Supplementary Table 1. Iron accumulation in tissues of FRDA patients

| Tissue | Number of FRDA subjects/cell lines studied^1^ (number of GAA repeats of allele1/allele 2) | Technique used for iron measurements | Findings | Reference |
| --- | --- | --- | --- | --- |
| Heart | 1 (NA)^2^ | Perls Prussian blue staining | Presence of iron pigment mixed with calcium | (Sanchez-Casis et al., 1976) |
| Heart | 3 (NA)^2^ | Perls Prussian blue staining | Dense intracytoplasmic deposits of iron particles | (Lamarche et al., 1980) |
| Heart  Liver  Spleen | Heart: 6 (780/-; 570/-; NA; NA; 370/-; 600/-)^3^  Liver: 2 (780/-; 570/-)^3^  Spleen: 2 (780/-; 600/-)^3^ | Perls Prussian blue staining | Iron staining in the three tissues, consistent with mitochondrial location in heart and liver | (Bradley et al., 2000) |
| Heart | 8 (952 ± 186/740 ± 195)^4^ | X-ray fluorescence | Highly localized, cytosolic increase of iron. | (Ramirez et al., 2012) |
| Heart | 4 (1050/700; 1200/1200; 694/593; 793/644) | Perls Prussian blue staining | Cardiomyocyte iron accumulation | (Huang et al., 2013) |
| Heart | 15 (718±159; 914±195)^4^ | X-ray fluorescence  Inductively-coupled plasma optical emission spectrometry | The in situ measurements revealed significant regional iron accumulation  Analysis of bulk extracts showed normal total iron levels | (Koeppen et al., 2015) |
| Heart | 23 (NA, all patients had homozygous GAA repeat expansions) | Inductively coupled plasma optical emission spectrometry  Single quadrupole inductively coupled plasma mass spectrometry | Total levels of iron in bulk extracts were higher than normal, but differences were not significant | (Kruger et al., 2016) |
| Heart | 4 (896/c.11_12TCdel; 744/exon 5 del; 1016/1016; 800/1100) | Perls Prussian blue staining | Presence of iron-containing inclusions | (Becker et al., 2017) |
| Dentate nuclei | 10 (783±357/609±306;  160-1122/106-1114)^5^ | X-ray fluorescence  Inductively-coupled plasma optical emission spectrometry | Increasing admixture of Cu, Zn, and iron signals | (Koeppen et al., 2012) |
| Dentate nuclei | 34 (1021, range 437–1705 / 878, range 437–1330) | Magnetic resonance imaging | Increased iron content | (Bonilha da Silva et al., 2014) |
| Dentate Nuclei  Midbrain (Red nuclei,  Substantia nigra)  Basal Ganglia (Caudate, Putamen, Pallidum)  Thalamus | 30 (548 ± 229/859 ± 255)^4^ | Magnetic resonance imaging | Increase iron content in the Dentate nuclei and Red nuclei and a trend increases in the Basal ganglia | (Harding et al., 2016) |
| Dorsal Root Ganglia | 4 (GAA repeats ranging from 242 to 1200)^6^ | X-ray fluorescence | Limited and regional increases of iron | (Koeppen et al., 2009) |
| Fibroblasts  Lymphoblast | 2 cell lines (658/1243; 753/820)  (NA) | Atomic absorption spectroscopy | A small increase in mean concentration of mitochondrial iron, but differences with iron content of controls were not significant | (Wong et al., 1999) |
| Fibroblasts | 4 cell lines (541/708; 596/1024; 537/812; 460/688) | Atomic absorption spectroscopy | Increase iron content in the mitochondrial fraction | (Delatycki et al., 1999) |
| Peripheral blood lymphocytes | 2 cell lines (600/W173G; 760/Y118stop) | Flame atomic absorption spectrophotometry | Higher ratio of mitochondrial filtrable: non-filtrable iron | (Tan et al., 2001) |

1, The number of control samples used in each studies has not been indicated in this table; 2, at that time the number of GAA repeats in the *FXN* gene was not known, because this gene was cloned in 1996; 3, the diagnosis of FRDA was based in clinical features and confirmed by the presence of a GAA repeat expansion in the *FXN* gene (Bradley et al., 2000); 4, mean ± standard deviation of GAA repeat number in allele1/allele2; 5, mean ± standard deviation and range of GAA repeats of the analyzed samples; 6, this study analyzed 19 FRDA patients in which the number of GAA repeats on the mutated alleles were indicated ranging from 242 to 1200 repeats. Among them, postmortem samples of DRG from 4 patients were used for high-definition X-ray fluorescence mapping of iron; NA, not available.

Supplementary Table 2. Iron accumulation in multicellular models of FRDA

| FRDA model (reference) | Description | Technique used for iron measurements | Tissue (number of samples studied)^1^ | Findings | Reference |
| --- | --- | --- | --- | --- | --- |
| MCK KO mice  (Puccio et al., 2001) | Muscle creatine kinase (MCK) conditional *Fxn* knockout (KO) mice  This mutant develops a severe cardiac phenotype  In the heart, frataxin expression is almost ablated | Atomic absorption spectroscopy  Perls Prussian blue staining | Heart (4) | Intramitochondrial iron accumulation at 10 weeks–old mutants, after pathology onset and inactivation of ISC enzymes | (Puccio et al., 2001) |
|  |  | Atomic absorption spectroscopy | Heart (5) | Mitochondrial iron accumulation occurs 4–5 weeks after the onset of heart pathology and ISC enzyme deficiency | (Seznec et al., 2004) |
|  |  | Inductively coupled plasma atomic emission spectrometry  Perls Prussian blue staining | Heart (27) | Fe levels were significantly greater in mutants than in wildtype mice | (Whitnall et al., 2008) |
|  |  | Perls Prussian blue staining | Heart (4) | Progressive iron accumulation from 5 to 10 weeks of age | (Huang et al., 2013) |
|  |  | DAB-enhanced Perls staining | Heart (3) | Mitochondrial iron accumulation | (Perdomini et al., 2014) |
|  |  | Perls Prussian blue staining | Heart (3-5) | Iron accumulation | (Anzovino et al., 2017) |
|  |  | Inductively coupled plasma atomic emission spectrometry | Heart  Liver  Kidney  Spleen  (28-37) | In all organs, iron levels of 10-week-old mutants were significantly greater than that of control mice | (Huang et al., 2009) |
|  |  | Inductively coupled plasma atomic emission spectrometry (ICP-AES)  Transmission electron microscopy, Mössbauer spectra and alternating current magnetic susceptibility | Heart  Liver  Kidney  Spleen  Serum  (24-30 for ICP-AES analysis;  4-10 to analyze the speciation and molecular form of Fe) | In all cases, Fe levels were significantly higher in normal and high-Fe-diet–fed mutants compared with wildtype at 8.5 week of age  Mitochondrial iron accumulation in the heart occurs as nonferritin mineral aggregates containing phosphorus and sulfur | (Whitnall et al., 2012) |
| YG22R mice  (Al-Mahdawi et al., 2006) | *Fxn* KO mice containing a *FXN* YAC transgene with approximately 190 GAA repeat expansion derived from FRDA patient DNA  These mice show mild FRDA phenotypes. They express only human frataxin, that its levels in the heart were calculated as 37% of physiological mouse frataxin level | Perls Prussian blue staining | Heart (8) | Iron deposition in the oldest (14–18 months) model mice | (Al-Mahdawi et al., 2006) |
| YG8R mice  (Al-Mahdawi et al., 2006) | Fxn KO mice containing a FXN YAC transgene with 190 + 90 GAA-repeat expansion derived from FRDA patient DNA  These mice show mild FRDA phenotypes. They express only human frataxin, that its levels in the heart were calculated as 25% of physiological mouse frataxin level | Perls Prussian blue staining | Heart (2) | Presence of abundant iron deposits in 18-month-old mutants. | (Rocca et al., 2017) |
| FRDAkd mice  (Chandran et al., 2017) | Transgenic mice containing inducible expression system delivering shRNA against frataxin  The *Fxn* knockdowns develop behavioral, physiological, pathological and molecular deficits that parallel the FRDA phenotypes  This mutant shows a high reduction of frataxin level | Gomori’s iron staining | Heart (3) | Substantially increased of iron deposits | (Chandran et al., 2017) |
| *Fxn^Alb^* mice  (Thierbach et al., 2005) | Albumin (Alb) conditional *Fxn* knockout (KO) mice  These mice show liver failure and premature death starting around 28 days of age (Martelli et al., 2015)  Frataxin expression is highly decreased in hepatocytes | Batophenanthroline-based spectrophometric  approach | Liver (6) | It is detected a 5-fold increase of mitochondrial iron level at 28, but not 14 days of age. At this time the activity of ISC enzymes is altered | (Martelli et al., 2015) |
| Prp-KO mice  (Simon et al., 2004) | Prion (Prp) conditional *Fxn* KO mice  Two lines were generated that develop a gradually progressive mixed cerebellar and sensory ataxia with loss of proprioception    Frataxin level is significantly  reduced in the cerebellum of both mutant lines, and in the brain of one line | Perls Prussian blue staining | Dorsal horn, Brain stem, Cerebellar granular layer,  Cortex  (4) | Very few iron deposits were seen in both mutant lines as a late feature in the disease | (Simon et al., 2004) |
| *Fxn*-sgRNAs mice  (Chen et al., 2016a) | This model has been obtained by using an adeno-associated virus and CRISPR/Cas9 strategy.  The animals have smaller body size, impaired locomotion and shortened life span  Cortical neurons show less than 40% of the *Fxn* mRNA of controls | DAB-enhanced Perls staining  RPA staining | Brain (3) | Fe^3+^ and Fe^2+^ levels accumulate both intracellularly and extracellularly | (Chen et al., 2016a) |
| *UAS-fhIR* (*Drosophila melanogaster*)  (Llorens et al., 2007) | This line has been obtained using the UAS-GAL4 transgene-based RNAi methodology to knockdown the *Drosophila* frataxin ortholog (*fh*) expression  Transgenic flies show decreased lifespan and develop impaired motor performance in adulthood  A reduction of up to 70% of frataxin expression is obtained using the actin-GAL4 driver at 25ºC | Atomic absorption spectroscopy | Whole organism | Total levels of iron were higher than normal, but differences were not significant  Mitochondrial iron content was significantly increased in the mutant flies  Increased levels of zinc, copper, manganese and aluminum | (Soriano et al., 2013, 2016) |
| *fh^1^* (*Drosophila melanogaster*)  (Chen et al., 2016b) | Mutant flies have a missense mutation (S136R) in *fh* induced by ethyl methanesulfonate compound  Hemizygous mutants are lethal in preadult phases  Severe loss of frataxin function | DAB-enhanced Perls staining  RPA staining | Larva brain, muscle, neuromuscular junctions, fat body, gut | Fe^+2^ and Fe^+3^ accumulations in multiple tissues | (Chen et al., 2016b) |
| *atfh-1* (*Arabidopsis thaliana*)  (Busi et al., 2006) | This mutant has a T-DNA insertion in the 5’-untranslated region of the *Arabidopsis* *thaliana* frataxin ortholog (*AtFH*) open reading frame  The mutant plants show retarded growth, reduced fresh weight of fruits and reduced number of seeds per fruit  Frataxin level is reduced more than 50% of the control level | Ferrozine method | Roots, flowers and leaves | The mutant line displays ~60% more total Fe/g of dry weight and ~ 20% more mitochondrial iron than wild-type | (Martin et al., 2009) |
| *as-AtFH* (*Arabidopsis thaliana*)  (Maliandi et al., 2011) | Transgenic plants that express a 564 bp *AtFH* fragment in antisense orientation under the control of cauliflower  mosaic virus 35S promoter  Transgenic plants show retarded growth at different developmental stages. Decrease of fruit fresh weight, alteration in silique length and a reduced number of viable seeds  Frataxin amount is reduced about 70% with respect to control levels | Ferrozine method | Leaves | Iron content increased ~40% in chloroplasts with respect to control plants | (Turowski et al., 2015) |
|  |  |  |  |  |  |

^1^ The number of control samples used in each study has not been indicated in this table. DAB, 3,3’-Diaminobenzidine; ISC, Iron Sulfur Cluster; RPA, Rhodamine B-[(1,10-phenanthrolin-5-yl) aminocarbonyl] benzyl ester.

**References**

Al-Mahdawi, S., Pinto, R. M., Varshney, D., Lawrence, L., Lowrie, M. B., Hughes, S., et al. (2006). GAA repeat expansion mutation mouse models of Friedreich ataxia exhibit oxidative stress leading to progressive neuronal and cardiac pathology. *Genomics* 88, 580–90. doi:10.1016/j.ygeno.2006.06.015.

Anzovino, A., Chiang, S., Brown, B. E., Hawkins, C. L., Richardson, D. R., and Huang, M. L.-H. (2017). Molecular Alterations in a Mouse Cardiac Model of Friedreich Ataxia: An Impaired Nrf2 Response Mediated via Upregulation of Keap1 and Activation of the Gsk3β Axis. *Am. J. Pathol.* 187, 2858–2875. doi:10.1016/j.ajpath.2017.08.021.

Becker, A. B., Qian, J., Gelman, B. B., Yang, M., Bauer, P., and Koeppen, A. H. (2017). Heart and Nervous System Pathology in Compound Heterozygous Friedreich Ataxia. *J. Neuropathol. Exp. Neurol.* 76, 665–675. doi:10.1093/jnen/nlx047.

Busi, M. V, Maliandi, M. V, Valdez, H., Clemente, M., Zabaleta, E. J., Araya, A., et al. (2006). Deficiency of Arabidopsis thaliana frataxin alters activity of mitochondrial Fe-S proteins and induces oxidative stress. *Plant J.* 48, 873–82. doi:10.1111/j.1365-313X.2006.02923.x.

Maliandi, M. V, Busi, M. V, Turowski, V. R., Leaden, L., Araya, A., and Gomez-Casati, D. F. (2011). The mitochondrial protein frataxin is essential for heme biosynthesis in plants. *FEBS J.* 278, 470–81. doi:10.1111/j.1742-4658.2010.07968.x.

Seznec, H., Simon, D., Monassier, L., Criqui-Filipe, P., Gansmuller, A., Rustin, P., et al. (2004). Idebenone delays the onset of cardiac functional alteration without correction of Fe-S enzymes deficit in a mouse model for Friedreich ataxia. *Hum. Mol. Genet.* 13, 1017–24. doi:10.1093/hmg/ddh114.

Thierbach, R., Schulz, T. J., Isken, F., Voigt, A., Mietzner, B., Drewes, G., et al. (2005). Targeted disruption of hepatic frataxin expression causes impaired mitochondrial function, decreased life span and tumor growth in mice. *Hum. Mol. Genet.* 14, 3857–64. doi:10.1093/hmg/ddi410.
